# Gastrointestinal Manifestations of Systemic Sclerosis

**DOI:** 10.4172/2161-1149.1000235

**Published:** 2018-03-30

**Authors:** Isabel M. McFarlane, Manjeet S. Bhamra, Alexandra Kreps, Sadat Iqbal, Firas Al-Ani, Carla Saladini-Aponte, Christon Grant, Soberjot Singh, Khalid Awwal, Kristaq Koci, Yair Saperstein, Fray M. Arroyo-Mercado, Derek B. Laskar, Purna Atluri

**Affiliations:** Division of Rheumatology and Gastroenterology, Department of Medicine and Pathology, Hospitals Kings County Hospital Brooklyn, State University of New York, USA

**Keywords:** Systemic sclerosis, Scleroderma, Gastrointestinal, Pathogenesis, Esophagus, Interstitial lung disease, Diagnosis, Management

## Abstract

Systemic sclerosis (SSc) is a rare autoimmune disease characterized by fibroproliferative alterations of the microvasculature leading to fibrosis and loss of function of the skin and internal organs. Gastrointestinal manifestations of SSc are the most commonly encountered complications of the disease affecting nearly 90% of the SSc population. Among these complications, the esophagus and the anorectum are the most commonly affected. However, this devastating disorder does not spare any part of the gastrointestinal tract (GIT), and includes the oral cavity, esophagus, stomach, small and large bowels as well as the liver and pancreas. In this review, we present the current understanding of the pathophysiologic mechanisms of SSc including vasculopathy, endothelial to mesenchymal transformation as well as the autoimmune pathogenetic pathways. We also discuss the clinical presentation and diagnosis of each part of the GIT affected by SSc. Finally, we highlight the latest developments in the management of this disease, addressing the severe malnutrition that affects this vulnerable patient population and ways to assess and improve the nutritional status of the patients.

## Introduction

Systemic sclerosis (SSc), is an autoimmune disorder, characterized by alterations of humoral and cellular immunity, leading to fibroproliferative alterations in the microvasculature which in turn causes excessive deposition of collagen fibers in the skin and internal organs [1,2]. This rare disorder affects the USA population with a prevalence of 276 persons per million and an incidence 20 new cases per million per year. In the USA, among those 35 to 64 years of age, SSc is four times higher in women compared to men [3–6]. In terms of racial differences, black women are the most severely affected, they tend to develop SSc at a relatively younger age particularly the diffuse subtype of the disease (dSSc) [7]. In the USA also, Choctaw Native Americans have the highest prevalence of SSc that is nearly double that of other ethnic groups [8].

Gastrointestinal (GI) involvement in SSc is almost universal affecting nearly 90% of the patients [2,9]. However, only 8% of the patients present with severe involvement leading to increased morbidity and mortality [10]. GI symptoms also could be the first manifestation of SSc in nearly 10% of the cases [11] and tend to occur equally in dSSc and lSSc populations [12]. The esophagus is the most commonly involved portion of the GIT followed by the anorectum and the small bowel [13]. Up to 50% of the patients with esophageal disease and 20% of those with small bowel involvement remain asymptomatic [9,14] because symptoms are often subclinical until severe tissue damage has taken place [15,16]. Five-year survival in SSc largely depends on the subtype of the disease and reported to be 80% in diffuse and 90% in limited categories. However, patients with GI involvement have significantly decreased survival that is reported to be 15 % at 9 years [10]. It is important to note that the development of malabsorption confers 50% increased mortality among SSc patients at 8.5 years [13,17].

## Pathogenesis of Scleroderma

The pathogenesis of SSc has generated a great deal of interest, currently several potential genetic and epigenetic triggers are being actively studied [18–21]. For example, Class I Human Leukocyte Antigen (HLA) allele types A, B, C, and G, as well as Class II HLA alleles DP, DQ, and DR have been found to confer susceptibility to SSc [18–21]. Furthermore, studies have shown that higher mortality in SSc is linked to HLA alleles DRB1*0802 and DQA1*0501 [22]. Among exogenous triggers, gadolinium, L-tryptophane, viruses and fungi are linked to SSc [23]. Furthermore, endogenous triggers, such as cancer, danger signals like Toll like receptor ligands and alterations in DNA and RNA, have also been postulated. DNA methylation, in particular hypomethylation can lead to overexpression of integrins which in turn favor myofibroblast differentiation and activation of transforming growth factor β (TGF-β) [24]. Micro-RNAs (miRNA) are involved in post-transcriptional regulation of gene expression and are different between those with limited and diffuse SSc spectrum of the disease. miRNA downregulation in SSc fibroblasts allowed decreased apoptosis and contributed to proliferative vasculopathy *via* increased urokinase plasminogen activity from the vascular smooth muscle cells [25].

### Vascular dysfunction and small vessel proliferation

**Figure 1:**
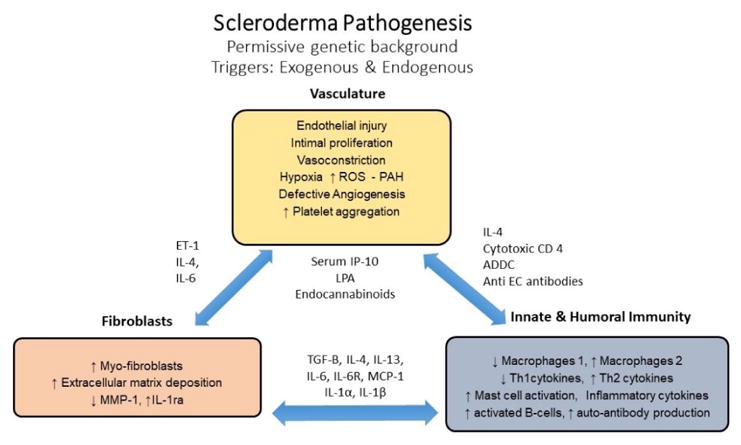
Pathogenesis of Systemic Sclerosis. ET: Endothelin; IL: Interleukin; ROS: Reactive Oxygen Species; PAH: Pulmonary Arterial Hypertension; CD: Cluster of Differentiation; ADCC; Antibody-Dependent Cellular Cytotoxicity; Anti-EC: Anti- Endothelial Cell; IP: Interferon-gamma Induced Protein; LPA: Lysophosphatidic Acid; MMP: Matrix Metalloproteinases; TGF-β Tissue Growth Factor β; MCP Monocyte Chemoattractant Protein.

Studies have shown that intimal proliferation occurs early in SSc and that it is possibly triggered by viruses, cytotoxic T cells, anti-endothelial antibodies. This intimal proliferation reflects either active or previous endothelial cell damage (Figure 1) [26,27].

Furthermore, ischemia-reperfusion injury manifested clinically as Raynaud’s phenomenon, leads to upregulation of adhesion molecules on endothelial cells which promotes leukocyte migration into tissues [27]. Migration of leukocytes into the local tissues can ultimately drive cell damage *via* production of reactive oxygen species. Reactive oxygen species are both directly toxic to the tissues but also dampen the release of vasodilatory mediators such as Nitric Oxide (NO) and prostacyclin [28–30]. Von Willebrand factor, a marker of endothelial injury, has also been found to be increased in the serum of SSc patients [28]. At the level of the sub-endothelium, the above described milieu favors platelet aggregation, fibrin deposition and intravascular thrombus formation [31]. In addition, defective angiogenesis plays a major pathogenic role causing drop out capillaries and changes in capillary architecture [27,32]. Studies have also shown that endothelial cell apoptosis is the likely culprit for capillary dropout and dermal fibrosis [33]. Anti-endothelial cell antibodies found in SSc serum were shown to induce endothelial cell apoptosis, release of cytokines, chemokines and increased expression of adhesion molecules [34]. Furthermore, endothelial to mesenchymal cell transition (EndoMT) is being increasingly recognized as driver of fibroblast/myofibroblast formation, rendering TGF-β, the main inducer cytokine followed by Interleukin-1β and Tumor necrosis factor-α [35]. In summary, vasculopathy leads to intimal proliferation and deposition of proteoglycan in the arterioles and capillaries resulting in fibrosis [36].

### Alterations of innate and adaptive immunity

While endothelial cell damage attracts inflammatory cells to perivascular tissue; macrophages and lymphocytes release cytokines that also promote recruitment of monocytes and stimulate perivascular fibrosis [37,38]. Other cytokines also play a role in the pathogenesis of SSC, for example, interleukin-13 (IL-13), *via* TGF-β production, renders macrophages (M2 macrophages) to become pro-fibrotic [39,40]. Furthermore, T cells infiltrating SSc damaged tissues produce cytokines and are implicated in the production of autoantibodies [41,42].

The role of TGF-β, connective tissue factor and insulin-like growth factor: Fibroblasts are key to the initiation and progression of fibrosis and control the extracellular matrix (ECM) structure by activating Matrix Metalloproteinase-1 (MMP-1) and Tissue Inhibitor of Matrix Metalloproteinases (TIMMP) [43]. ECM alterations caused by tissue injury drive migration and attachment of fibroblasts and its transformation into secretory myofibroblasts (Figure 1). However, myofibroblasts can arise not only from resident fibroblasts but also from different cell sources such pericytes, smooth muscle cells, and epithelial cells [44]. Studies have shown that Interleukin-1α and IL-1β levels promote not only the proliferation and survival of cultured fibroblast but also increase the production of TIMMP, collagen inhibitors, MMP-1 and hyaluronan, suggesting that IL-1 favors the longevity of myofibroblasts in SSc [45,46].

CD4^+^ and CD8^+^ T cells play an important role in the pathogenesis of SSc (Figure 1). They belong to a type 2 phenotype with the ability to produce IL-13. While CD8^+^ T cells are most abundant in the skin during the early stages of SSc, and CD4^+^ are found in late-stages. Both types of T cells are able to produce IL-13 [44]. Lack of suppression by dysfunctional T-regulatory cells also favors fibrosis, which reflects alteration in the homeostasis of the immune system [47,48].

## Gastrointestinal Changes in Scleroderma: Pathogenetic Insights

**Figure 2:**
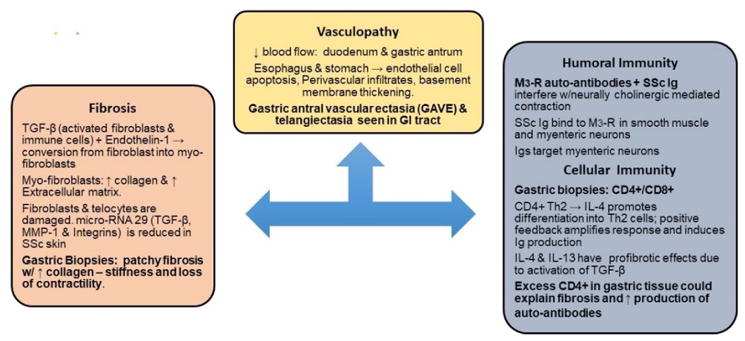
Pathogenesis of Gastrointestinal Involvement in Systemic Sclerosis.

Parallel to those occurring in systemic scleroderma, GI manifestations include vascular changes, alteration of innate immunity as well as the process of fibrosis (Figure 2).

### Vascular changes

As discussed above endothelial cell damage results in exposure of adhesion molecules and production of reactive oxygen species which dampen vasodilation. This milieu promotes ischemia and recruitment of inflammatory cells and M2 macrophages that promote fibrosis on the extracellular matrix [49]. The above described pathogenesis does have distinct tropism including the skin as well as to the GIT [50]. Vascular changes in SSc were demonstrated by video capsule endoscopy. In a study of 50 SSc patients gastric mucosal damage was seen in 86.7% that included watermelon stomach, angiodysplasia and telangiectasia. In addition, the small bowel from duodenum to ileum was affected in 50% of the cases [51].

Alterations of innate and adaptive immunity: While early studies highlighted the utility of SSc antibodies as markers of disease severity, these antibodies were not considered to play any role in the pathogenesis of this disease [52,53]. Currently, these antibodies, among other autoantibodies, have been linked to disease complications such as pulmonary hypertension, renal failure and abnormalities in GI motility [52,53]. In normal individuals, action potentials traveling along myenteric neurons induce the release of acetylcholine (Ach) into the synaptic cleft of the neuromuscular junction. Ach binds to Muscarinic 3 receptor (M3-R) located at motor end plate of smooth muscle cells promoting muscle contraction. Ach also binds to M3-R located in the myenteric neuron to autoregulate its own release. In SSc, Ach’s function on GI motility is affected by pathogenic antibody production (Figure 2) [54,55].

Anti-myenteric antibodies were described and linked to pathogenesis of SSc [54]. Studies demonstrate that M3-R autoantibodies in SSc cause dysfunction *via* a neural as well as a myogenic effect [55–57]. Neuropathic damage occurs early in SSc, *via* IgG autoantibodies to M3-R at the myenteric neuron preventing the autoregulatory release of Ach from neurons [54]. This was demonstrated in SSc patients by studying the esophageal smooth muscle contractile response to the direct acting-muscarinic agonist methacholine versus edrophonium, which acts indirectly preventing the released Ach from being metabolized by Acetylcholinesterase [58]. In 2016, *in-vitro* studies showed potential reversible changes to SSc-associated intestinal dysfunction at both the neuropathic and myopathic stages with IVIG therapy [59]. Of note, reversible changes with IVIG were only seen prior to profound tissue atrophy seen in later-stage SSc. Cell mediated immune dysfunction in SSc comes mainly from Type 2 helper (Th2) CD4^+^ T cells (Figure 2) [60]. Th2 cytokines interleukins (IL) IL-4 and IL-13 induced fibroblast activation, whereas the Th1 cytokine interferon γ (IFN-γ) blocks cytokine-mediated fibroblast activation [52]. Furthermore, IL-4 amplifies its own response and up-regulates humoral immunity by inducing immunoglobulin production and isotype switching (Figure 2).

The immunology of Th2 CD4^+^ T lymphocytes in SSc were implicated in the pathogenesis of fibrosis when several gastric biopsy specimens demonstrated significant tissue infiltration [61]. CD4^+^ cells have been shown to induce fibrosis *via* the release of profibrotic cytokines and direct effect on fibroblast [52].

### Fibrosis

The pathogenesis of fibrosis is paramount as it is the fibrosis that portends profound morbidity and mortaility in SSc patients. Proliferation of Th2 cells in the local tissue is also involved in fibrosis as it leads to an increase in TGF-β production. TGF-β produced by activated fibroblasts and immune cells leads to extensive fibrosis and participates in the conversion of fibroblasts into myofibroblasts (Figure 2) [62,63].

Myofibroblasts subsequently produce excess type I and type III collagen. Changes in the gastric wall of SSc individuals show severe fibrosis, different collagen types, myofibroblast, and abundant expression of several profibrotic factors (Figure 2) [64]. The end result from the altered tissue meshwork is impaired neuronal signal propagation and tissue contraction. These specific changes contribute to the previously mentioned autonomic dysfunctions and GI dysmotility [65].

## GI Involvement in SSc

As mentioned above, the manifestations in SSc involves nearly every part of the GIT, however, the affected regions vary in frequency and severity of involvement. Therefore, we will discuss each section of the GI involvement separately.

### Oral Cavity

Scleroderma within the oral cavity may present with a diverse range of manifestations. Microstomia (decrease in mouth aperture) and microcheilia (decreased mouth width) are common manifestations of SSc affecting 50 to 80% of the patients [66–68]. Microstomia is caused by fibrosis of the perioral tissues which can affect speech, mastication, and predispose these patients to oral and dental disease. Microstomia portends profound morbidity on a psycho-social level [68]. Increased levels of anti-topoisomerase I antibodies were found to correlate with the severity of microstomia encountered among SSc patients [69].

Xerostomia, is reported in up to 30–40% of SSc patients [66,67]. In a study of labial salivary gland biopsies in SSc patients with xerostomia, 58% of the cases had glandular fibrosis and 23% met criteria for concurrent Sjogren’s syndrome (SS). The subgroup of patients with SSc and SS were found to have lSSc at a significantly higher frequency [70]. Xerostomia predisposes to increased dental caries, difficulties in wearing prostheses, altered taste sensation, burning mouth syndrome, mucosal atrophy and candidiasis [68,71]. The teeth of SSc patients can be affected directly or indirectly by a number of factors. Saliva with low pH (xerostomia and esophageal reflux) leads to a direct damage to the enamel. Moreover, microstomia and hand deformities can limit proper oral hygiene further promoting poor oral health [68]. Widening of the periodontal ligament space (PLS) is a common radiographic finding among patients with SSc with the premolar and molar area being the most commonly involved [72]. Postulated mechanisms for PLS widening include higher resistance to mouth closure *via* masticator muscle involvement, primary trauma of the tissue, or excess collagen deposition [73]. Research does not support a direct correlation of PLS widening and its clinical impact on oral health [69]. Therefore, dentists taking care of SSc patients must be aware of these findings as to not take unnecessary invasive measures [68].

Management of xerostomia includes frequent water sips, artificial saliva throughout the day, special toothpastes, mouthwashes, and avoidance of medications and other therapeutic agents that may exacerbate xerostomia such antihistamines, antispasmodics, and tricyclic antidepressants among others [67,68]. Therapies proven helpful in managing early stages of microstomia include stretch mouth exercises, connective tissue massage, Kabat technique and kinesiotherapy [74]. Finally, hygienic dental practices including regular evaluation by a dental hygienist is highly recommended for patient-specific and tailored management.

Mandibular erosions at the condyles and coronoid processes, points of insertion of the masticatory muscles, have been described among SSc patients [72]. Vasculopathy affecting the small arteries of the mandibular bone along with pressure ischemia due to skin tightening results in increased mandibular resorption in SSc [75]. Temporomandibular joint (TMJ) involvement leads to pain and difficulty in mouth opening, symptoms that correlate with the length and involvement of the disease [76]. Panoramic dental imaging can also detect mandibular resorption among SSc patients (10–20%), condition that carries increased likelihood for pathological fractures, development of osteomyelitis and trigeminal neuralgia [77].

Patients with dSSc have a 25-fold increased risk of developing squamous cell carcinoma of the tongue [78]. Early diagnosis and tissue sampling is challenging in SSc due to the reduced oral aperture and changes in surrounding tissues. In addition, 25% of SSc patients can develop oropharyngeal dysphagia, which may arise not only from muscle damage to the lower pharynx and superior one third of the esophagus but also as part of gastroesophageal reflux (GERD) [79].

In order to assess the degree of impairment and impact on the quality of life due to oral cavity involvement in SSc, a number of tools have been developed. The Oral Health Impact Profile is the most widely used [80]. However, the Mouth Handicap in Systemic Sclerosis (MHISS) scale is reliable, has good construct validity, and assesses mouth-specific disability in SSc patients [81].

### Esophagus

**Figure 3:**
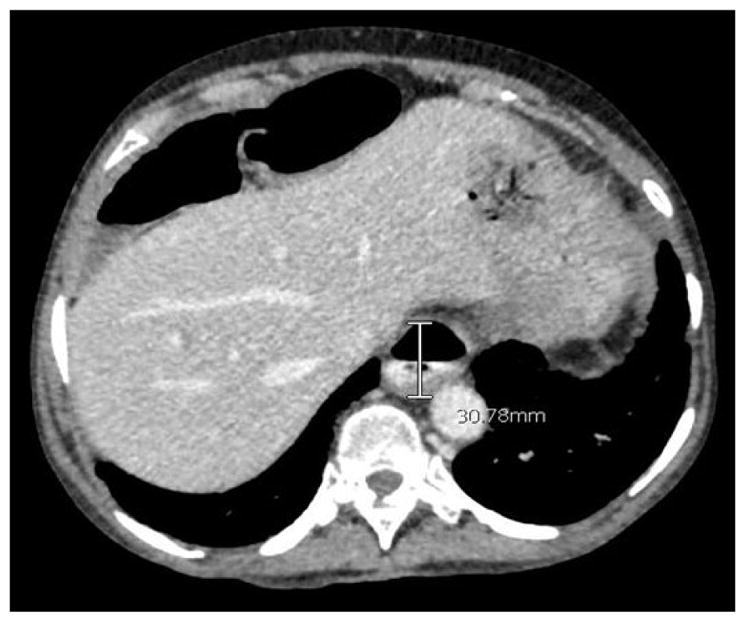
CT Abdomen with oral contrast in a patient with SSc demonstrates profound dilation of the distal lower third of the esophagus with 30 mm dilation and layer of intraluminal contrast.

The esophagus is most commonly affected organ of the GIT [82]. Patients could present with dysphagia, odynophagia, heartburn, regurgitation, chronic cough, or hoarseness [83,84]. These symptoms are largely the result of structural and functional changes in the esophagus including reduced lower esophageal sphincter (LES) pressure, presence of hiatal hernia, low or absent peristalsis and sicca syndrome [85]. Dilation can be seen along the entire esophagus with some studies showing a mean dilation of 14.7 mm [86]. However, more severe dilatation could occur, Figure 3, as was observed in a woman with a 20-year history of lSSc. The esophagus can be involved in both limited and diffuse SSc, however studies suggest lesser esophageal involvement in lSSc [87]. The prevalence of reflux, dysphagia, and adenocarcinoma has been reported as high as 34.8%, 4.3%, and 1.9% respectively in a large cohort of patients with SSc [82]. The pathogenesis of esophageal involvement includes microvascular and inflammatory changes of the esophageal smooth muscle, nerves, and connective tissues which contribute to fibrosis and ultimately to esophageal dysfunction [82,88,89].

**Figure 4:**
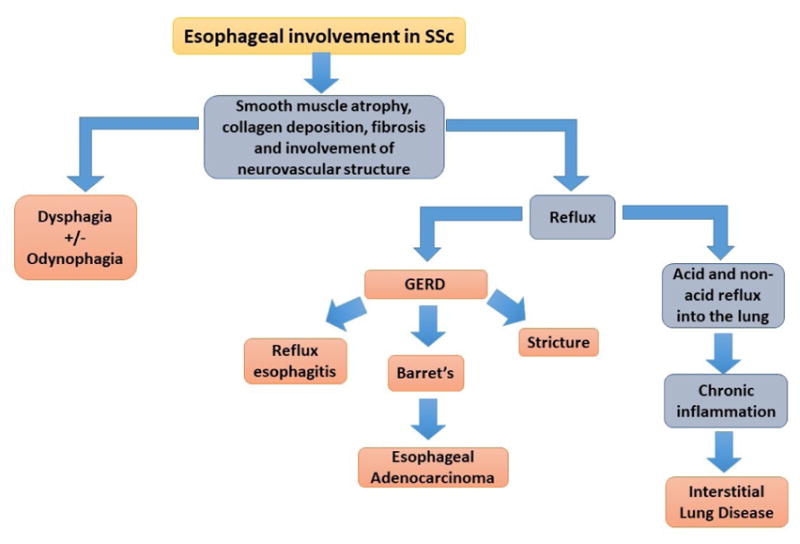
Summary of pathogenesis of ILD and relationship to GERD.

Decreased or absent peristalsis in the lower two thirds of the esophagus predisposes SSc patients to gastroesophageal reflux disease (GERD). The reduction in LES tone favors chronic reflux changes, which can lead to other complications such as reflux esophagitis, strictures and Barrett’s esophagus (Figure 4) [82,90].

The management of GERD includes dietary and lifestyle interventions such as avoidance of aggravating foods, alcohol, tobacco products, and having meals within three hours from bedtime [91]. A daily proton pump inhibitor (PPI) is given for moderate to severe GERD. Specific strategies used for resistant patients include trails with different PPIs, dose increases, as well as PPI doses taken specifically 30–60 min before meals [91]. If the patient is already on PPI twice a day but symptoms persist, the addition of nightly histamine2 (H2)- blocker or pH impedance testing could be considered [91]. H2- blockers are effective given that nocturnal acid production is largely dependent on histamine release [91]. However, the use of antihistamines should be weighed appropriately in individuals with profound xerostomia as mentioned previously. Prokinetic agents have been reported to improve GERD symptoms but clinical trials have failed to show success with these agents [91]. PPIs and prokinetic therapy may be helpful in early esophageal disease however, these drugs lose efficacy in advanced disease when esophageal atrophy is present [91]. Surgical interventions such as fundoplication are only reserved for severe cases although the results might be less than desirable [86,90,91]. Effective disease management of GERD in systemic sclerosis often involves a multidisciplinary approach including rheumatologists, gastroenterologists and nutritionists [91].

Dysphagia to solids often leads the clinician to consider esophageal strictures [92]. Strictures are often a complication of poorly controlled chronic GERD [91]. Treatment often involves optimization of GERD therapy to prevent recurrence and worsening of strictures however, if the patient remains symptomatic, endoscopic dilation is an option [91]. In more complicated cases (such as asymmetry, diameter <12 mm or inability to pass endoscope), repeated dilations may be indicated. If dysphagia to solids and liquids is present, GERD, candida esophagitis and pill-induced esophagitis must be considered [92]. Candida esophagitis could be secondary to immunosuppression or acid suppression from PPI [92]. One study found that 15% of patients with SSc had hyphae on biopsy smears obtained during EGD [92,93].

**Figure 5:**
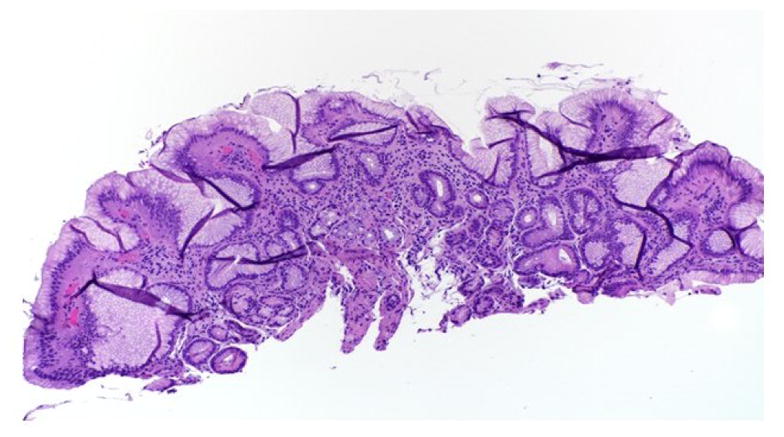
Low-power view shows columnar-lined epithelium, gastric pits, and underlying mucous glands with scattered chronic inflammatory cells within the underlying lamina classically seen with Barrett’s changes.

Additionally, patients with SSc are more likely to have uncontrolled GERD, esophagitis and higher risk for developing Barrett’s esophagus [91]. Barrett’s esophagus involves a change from normal squamous epithelium to specialized columnar-lined epithelium in the esophagus; this conversion is a major risk factor for esophageal adenocarcinoma (Figure 5) [91]. A three-year follow-up study to evaluate the progression of Barrett’s esophagus, showed a risk of 3% per year of converting from Barrett’s to high grade dysplasia or esophageal adenocarcinoma [94]. Screening measures for Barrett’s rely on the detection of dysplasia and the characterization of low grade versus high grade dysplasia [91]. For Barret’s esophagus without dysplasia, screening could be performed every 3–5 years, for Barrett’s with low grade dysplasia every 6–12 months and for cases with high grade dysplasia the recommendation is every 3 months. In the past, surgery was the only option for dysplasia but now with the advent of radiofrequency ablation and other advanced endoscopic techniques, the recommendation is for endoscopic eradication over surveillance for high grade dysplasia [91]. Given the lack of benefit for PPIs in long term prospective trials, the American Gastroenterology Association recommends against using PPIs for the sole reason of reducing cancer risk and disease progression when patients have Barrett’s esophagus [91].

### Esophageal involvement and interstitial lung disease

As we have previously mentioned, esophageal involvement can remain subclinical in early SSc. Despite lacking reflux symptoms, SSc patients show esophageal dilatation on chest tomography and esophageal abnormalities on endoscopy in the range of 30% to 75% of the cases [95–97]. Subclinical involvement of the esophagus in SSc has gained attention due to mounting evidence linking esophageal involvement with the development of interstitial lung disease (ILD).

ILD in SSc portends profound morbidity and mortality [14,90]. To date, there is not a thorough understanding of how ILD develops however, current data suggests that esophageal disease may independently contribute or be a source of ILD in SSc [90,98]. Observational studies, utilizing diffusion capacity of the lung for carbon monoxide (DLCO) to estimate progression of ILD, have shown that SSc patients with more active reflux disease ultimately develop more advanced ILD [98]. A retrospective review that included more than 400 SSc patients demonstrated that a wider diameter of the esophagus correlated with reductions in DLCO and FVC [14,90,98]. It is not known, whether particulate reflux or acid reflux or both are involved in the development of ILD. However, one study identified similar levels of pepsin in the bronchial and gastric fluids in SSc patients [99].

### Stomach

The two most common manifestations of SSc in the stomach are gastroparesis and gastric antral vascular ectasia (GAVE) resulting from neuropathic damage and vasculopathy respectively. GAVE can present as iron deficiency anemia due to chronic bleeding or emergently as an acute GI bleed [92]. On upper endoscopy, GAVE manifests as multiple parallel longitudinal columns of red vessels giving the characteristic “watermelon stomach” appearance. GAVE pathophysiology is very similar to the immune-mediated vasculopathy found in telangiectasias or Raynaud’s phenomenon [100]. Prevalence of GAVE is estimated to be anywhere between 5.7–22.3%. While GAVE usually presents in the first few years of established disease, it could also be the initial SSc feature. Therefore, evidence of GAVE on endoscopy should be followed by a comprehensive clinical evaluation, autoantibody testing and nail-fold capillaroscopy to assess for SSc [100,101]. There seems to be no difference between limited and diffuse SSc regarding their association with GAVE however, a study did show earlier development of GAVE and more severe anemia in dSSc [100,102–104]. The lack of association of GAVE with anti-topoisomerase I antibodies has been proven in multiple studies but the association of anti-RNA pol III (anti-RNP III) autoantibodies with GAVE is still unclear. Initial therapeutic measures for GAVE are often conservative, including iron supplementation and monitoring of anemia; volume repletion with intravenous fluids and blood products will be necessary in the case of GI bleed. If conservative therapy fails, then endoscopic therapy would be required with laser photocoagulation, argon plasma coagulation or endoscopic band ligation [101–103]. The usefulness of radiofrequency ablation (RFA) therapy has also been evaluated to treat GAVE cases after APC failure [105,106]. Surgical antrectomy is only considered when all other therapeutic measures have failed [91].

Gastroparesis occurs in 50% of SSc patients based on the diagnostic methodology used [107]. The pathophysiology of gastroparesis in SSc is multifactorial and presumed to occur in a stepwise fashion, beginning with vascular damage, followed by neurogenic impairment and finally myogenic dysfunction [92]. Once mechanical obstruction has been ruled out, gastroparesis can be confirmed by prolonged gastric emptying on imaging or antroduodenal manometry [91]. Symptoms of gastroparesis include but are not limited to early satiety, bloating, nausea, vomiting [92]. GERD aggravates gastroparesis and therefore GERD management should be optimized for the gastroparesis patients [91,100]. Prokinetic agents, such as metoclopramide, domiperidone, macrolide antibiotics among others, are helpful early in SSc, although adverse drug effects such as tardive dyskinesia, prolongation of QTc interval, and tachyphylaxis could portend significant mortality [91]. At later disease states, gastroparesis may prove too difficult to medically manage and percutaneous gastrostomy tube may be an option [92]. Immunosuppressants have not been proven to arrest the progression of GAVE in SSc [108].

Dietary modification such as small frequent meals with high content in soluble fibers, avoidance of fatty foods is also recommended [109]. Non-conventional modalities such as acupuncture have been shown to provide some improvement in GI symptoms and functioning in SSc patients specifically. If gastrostomy tube cannot be placed (due to non-functioning small bowel), parenteral nutrition may be needed [91]. Surgical interventions such as gastrectomy tend to carry even a higher risk of complications and are usually offered as last resort [110].

### Small Bowel

The small bowel is the second most common organ affected in SSc. The duodenum is the most frequently affected portion of the small bowel with a prevalence that ranges from 40–88% [111]. Over 65% of SSc patients with small bowel involvement remain asymptomatic [11,65]. Clinically, small bowel involvement in SSc can manifest as dysmotility due to stiffness and poor smooth muscle activity, chronic intestinal pseudo-obstruction (CIPO), pneumatosis cystoides intestinalis, small intestinal bacterial overgrowth (SIBO) and jejunal diverticula [100].

Dysmotility, the hallmark of SSc in the GIT, may be due in part to both neuropathic and myopathic changes. Vascular ischemia can cause nerve damage, smooth muscle atrophy, and subsequent fibrosis all of which contribute to the dysmotility observed in SSc [111]. The physiologic Migrating Motor Complexes (MMC) are considered abnormal in over 88% of SSc patients [112]. Interrupted or slowed propagation, decreased frequency, reduced contraction amplitude (in almost all patients) has been described. However, MMC defects in SSc do not correlate with symptoms [113].

Impaired motility may induce stasis of small intestine content and enhance upstream bacterial colonization, contributing to SIBO syndrome. SIBO affects between 43–56% of SSc patients [114]. Unlike motility itself, severity of bacterial overgrowth may correlate with symptoms, which may include nausea, vomiting, diarrhea, abdominal distension, bloating. Patients often have signs related to malabsorption, including weight loss, steatorrhea, vitamin and nutritional deficiencies [114].

SSc patients can also develop multiple air-filled cysts within the intestinal wall, a condition called pneumatosis cystoides intestinalis (PCI). PCI is a rare radiographic finding characterized by radiolucent cysts due to the presence of air in the submucosa or sub-serosa [115,116]. The development of PCI has been attributed to bacterial overgrowth. Excess gas production from bacterial overgrowth results in elevated intra-luminal pressures favoring the migration of gas into the intestinal wall. The coalescence of gas in the intestinal wall gives rise to the characteristic PCI appearance on imaging. Ruptured intraluminal cysts may result in benign pneumoperitoneum [116,117]. Benign spontaneous pneumoperitoneum in SSc should be treated conservatively with oxygen, antibiotics and bowel rest [65].

Chronic intestinal pseudo-obstruction (CIPO) is a syndrome characterized by impaired gastrointestinal propulsion with symptoms and signs of acute or chronic bowel obstruction in the absence of any mechanical occlusion of the gut lumen [118]. Chronic pseudo-obstruction can be secondary to atony, dilatation, and delayed transit within the small bowel. Perforation may also occur due to serosal fibrosis with loss of wall compliance in the muscularis layer. Lastly, jejunal diverticula may develop due to intestinal wall protrusion at the points of muscularis atrophy and fibrosis [100].

Several evaluations may be performed to evaluate the extent of small bowel involvement. Barium studies may show dilated loops of bowel with evidence of “flocculation” as well as pooling of the material [112]. In addition, tightly packed valvulae conniventes, thickened folds or what is known as “hide bound” bowel may be seen [112]. Alternative methods to visualize defects of the small bowel include scintigraphy, wireless motility capsule, and CT/MRI enterography [114]. If SIBO is suspected, a glucose hydrogen test or methane breath test may be performed. On jejunal aspirate, ≥ 105 organisms may be found. Anemia may be seen on laboratory studies, low vitamin B12 (due to microbial consumption) and high folate levels (due to bacterial production) [119]. Other significant findings include low albumin, iron, carotene, and selenium, indicative of nutritional deficiencies. Carbohydrate fermentation may also occur, causing lactic acidosis syndrome. Findings will include elevated lactic acid in the blood and D-lactic acid in the blood and/or urine. This may delineate bacterial overgrowth from other metabolic deficiencies [114].

Workup for bacterial overgrowth should also include stool cultures and analysis of stool to detect acidity and reducing substances or unabsorbed sugars [112]. Small bowel manometry may show uncoordinated and minimal MMC activity during fasting and post-prandial phases. These findings are most significant in post prandial phase and during fasting, where uncoordinated as well as minimal motor activity may be detected in the small bowel [92].

Therapy is targeted at symptomatic relief. The main approach for SIBO involves the use of antibiotics including amoxicillin, ampicillin, rifaximin, tetracycline and doxycycline. Antibiotic therapy may vary depending on symptom remission and degree of dysmotility. Certain patients may only require a five to seven-day course, while others a more prolonged regimen due to persistent diarrheal symptoms. With recurrent SIBO, patients may require intermittent courses of antibiotics. Some patients are placed on a schedule in which they may take antibiotics the first few days of every month or every other week [65]. The antibiotic regimen should be alternated to prevent resistance and relapse. In addition, metronidazole may be added if diarrhea persists despite treatment modifications. For pseudo-obstruction and dysmotility, prokinetics may assist in both acute and chronic cases. Medications including metoclopramide, domperidone, octreotide and cisapride may be added to assist with duodenal contraction and transit. Lastly, octreotide in combination with erythromycin has been found to also be very effective [120]. However, prolonged use of octreotide could lead to cholelithiasis and bowel perforation [121].

Diet may play an integral role in maintaining adequate motility. For constipation, adequate intake of fluids is crucial. Patients should avoid high fiber foods and laxatives, which may worsen the condition. In regard to diarrheal symptoms, foods containing lactose should be avoided. Studies have also shown a correlation between gastrointestinal disease severity and nutritional status, highlighting the importance of controlling GI symptoms to prevent malnourishment [88]. Total parenteral nutrition (TPN) may be crucial when other approaches have failed to relieve pseudo-obstruction [122]. TPN may correct malnutrition and improve quality of life long term for patients. In extreme cases of gastric dysmotility with preserved intestinal motility, a jejunal feeding tube may be placed for nutritional support [123].

Surgery is not often warranted, though it may be life-saving in cases of bowel perforation and ischemia [65]. Surgery must be conservative, paying particular attention to preserving the ileocecal valve and preventing prolonged ileus from bowel manipulation.

### Colon and Anorectum

Colonic involvement affects 20–50% of SSc patients. Although the majority of patients are asymptomatic those with long-standing disease have more severe symptoms. In addition, patients with cardiac, pulmonary, renal, and skin involvement tend to have more GI symptoms including diarrhea, constipation and a decreased gastro-colic reflex [65].

Colonic symptoms are a direct manifestation of colonic dysmotility and muscular atrophy [124]. It is postulated that inflammation in the intestinal wall can lead to fibrosis, muscular atrophy, and ultimately dysmotility. In severe cases, mucosal myoelectric recording showed almost absent myoelectric activity [124]. Similarly, to patients with diabetes mellitus and amyloidosis, progressive SSc patients have a significant decrease in the gastro-colic response to eating [124]. The pathology observed in the gut wall has been understood to have a neurologic component. One study demonstrated that contractility could be induced with direct exogenous stimuli (neostigmine) but not induced with indirect neural modulators [124]. Ultimately, this suggests that the nerves themselves are involved in the pathology. In the setting of smooth muscle atrophy seen in the later stages, it is not expected to see gut wall motility to return even with direct stimulation [100].

Colonic hypomotility can cause delayed gut transit time and subsequently constipation [65,125]. Constipation potentiates bacterial overgrowth which can ultimately lead to a malabsorptive diarrhea [65,126].

**Figure 6:**
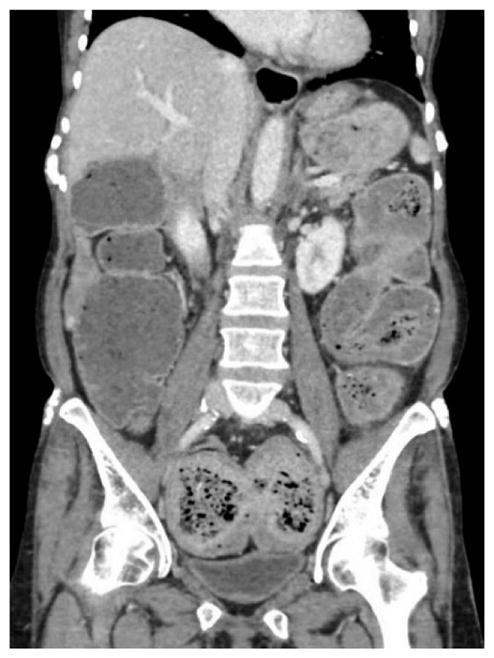
Loss of haustra due to weakened wall integrity noted in the ascending limb of the large bowel.

**Figure 7:**
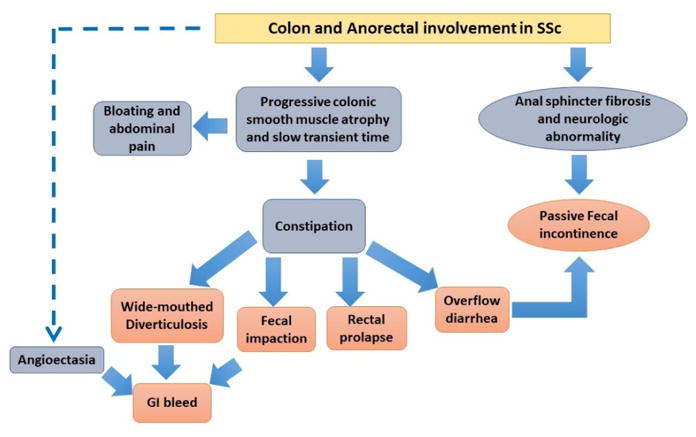
Colon and anorectal involvement in Systemic Sclerosis.

Diverticulosis affects the colon of SSc patients. Atrophy of the smooth muscle in the colonic wall causes dilatation and loss of haustras leading to “wide-mouth” diverticuli [127]. The Figure 6 demonstrates the loss of haustra in the ascending limb of the large bowel. These “wide-mouth” diverticuli can become ulcerated, infected, or even perforate from fecal impaction. In the later stages of the disease when the intestinal wall becomes rigid, a pseudo-resolution of the diverticuli could be apparent (Figure 7). has an overview of the multitude of symptoms present in SSc and a rough order of when they would present in the natural disease course.

Chronic intestinal pseudo-obstruction (CIPO) is a rare complication of SSc manifesting as episodic bowel obstruction without any mechanical component despite signs and symptoms of a true bowel obstruction [126]. Patients usually experience nausea, vomiting, abdominal distention, and changes in bowel habits. CIPO is the result of severely impaired contractility of the large bowel [126]. Therefore, when the clinical picture is suggestive, CIPO should be evaluated with radiographic and manometric modalities [127]. Surgical intervention would be only last resort for CIPO cases resistant to conservative management and to address complications such as bowel perforation [127].

New onset constipation in a SSc patient requires endoscopic and/or imaging studies to exclude underlying malignancy, strictures, diverticulosis and other pathology affecting the colon [126]. The American Society of Gastrointestinal Endoscopy recommends colonoscopy in patients with constipation if they have alarm features (weight loss, rectal bleeding, heme-positive stool, obstructive symptoms, rectal prolapse, or age >50 years); these alarm features are almost always seen in SSc patients [128]. Unfortunately, no definitive therapeutic modalities are available for colonic involvement. Treatment is directed toward symptomatic relief and the prevention of possible complications [126].

Supplemental fiber is a reasonable option in mild cases of constipation but unlike other types of constipation, fiber supplementation has three main limitations in SSc. Firstly, SSc patients suffer from gastroparesis and fiber supplements can worsen their symptoms. Secondly, fiber itself can worsen bloating and flatulence because fiber slows the gut transit time. Lastly, in severe cases supplementing fiber can lead to fecal impaction [129,130].

Regarding the role of laxatives, stimulant laxatives like senna and bisacodyl are usually preferred over osmotic laxatives like lactulose. Lactulose is only recommended for short periods due to its tolerability and the need for dose escalation; osmotic laxatives can worsen preexisting electrolyte imbalance from underlying renal disease [91,129]. Among the promotility agents, metoclopramide is a reasonable option that can be considered for long term use if no neurological side effects are observed [124]. Other medications shown to be effective include domperidone and prucalopride, but they are not approved in USA [131,132].

Surgery is deemed last resort in the management of CIPO as prolonged postoperative ileus can hamper the recovery process. Colonic resection is only recommended in patients with severe pseudo-obstruction and perforation. A novel emerging therapy is electrical pacing. It has shown to improve dysmotility and transit time in patients with constipation [65,133].

In SSc, anorectum is the second most commonly affected organ in the GIT after esophagus. About 50–70% of the patients with SSc reports to have anorectal symptoms. Multiple studies have measured different anorectum elements-tone, local reflex, sensation, and motility. In most of cases, the internal anal sphincter (IAS) is affected leading to symptoms such as fecal incontinence, constipation, and rectal prolapse [134].

Almost 24% of the patients fail to report fecal incontinence due to the distressing nature of this condition [135]. Fecal incontinence in SSc arises from structural and neurogenic alterations. Initially, structural changes were attributed as the cause for fecal incontinence since the internal anal sphincter (IAS) of SSc patients is thinner than those of healthy individuals. So, IAS atrophy was considered the basis for fecal incontinence [136]. However, imaging studies have revealed that these thinner IAS have slower gadolinium-enhancement suggesting vascular insufficiency. Hence, vasculopathy was proposed to play an instrumental role in the functional and structural impairment of the IAS [137]. This finding resembles the vascular insufficiency that was observed in SSc patients with decreased esophageal tone [135]. In addition, IAS dysmotility may be due to a primary muscle or neuron pathology as opposed to a secondary process involving ischemia or mural thickness inflammation [138]. These hypotheses are based on several studies which have complied findings including abnormal neuron conduction, depletion of neurotransmitters, and perineural mast cell proliferation on deep rectal tissue biopsies in patients with lSSc [139]. Moreover, the primary neuron abnormalities are seen in the tissue prior to fibroblast proliferation and collagen deposition in the GIT. Decreased recto-anal inhibitory reflex is further evidence of local neurologic impairment in SSc [140]. It paradoxically causes fecal incontinence as the local reflex system fails to relax in response to distention. Resting capacity and pressure of the sphincter were also found to be reduced however, no significant changes were noted in rectal tone or sensation [140].

Constipation and rectal prolapse occur as the disease progresses. Although diminished sphincter function can cause constipation, fibrosis and muscular atrophy causes impaired relaxation and restricted distention [141]. This leads to constipation and fecal impaction. Moreover, weakness of the submucosa and chronic straining of the rectal wall and IAS can cause rectal prolapse. Pressure of the rectal prolapse can further decrease the pressure of IAS, causing fecal incontinence [126]. Manometric studies allow the detection of reduced anal sphincter resting pressure and compliance, with impaired rectoanal inhibitory reflex and normal squeeze pressures [142]. Anal sphincter *via* endoanal ultrasound (EAUS) or rectal magnetic resonance imaging can also assess the integrity as well as structural abnormalities [135,143].

Conventional therapies such as diet, stool bulking agents and medication can be useful if diarrhea or loose stool is present. Diet modification can mediate gut motility and integrity of the IAS, improving fecal incontinence. Anti-diarrheal medication can be helpful, but they need to be used carefully as they can exacerbate constipation and induce rectal prolapse [65]. Recently, sacral nerve stimulation is being studied in SSc patients with fecal incontinence. Although patients with fecal incontinence benefit from sacral nerve stimulation therapy, it does not provide long term benefits to SSc patients [144,145]. However, a randomized placebo-controlled study published in 2014 has suggested that posterior tibial nerve stimulation could be an alternative effective method of neuromodulation and relief of fecal incontinence symptoms in SSc [146]. Biofeedback is another therapy that can be beneficial to patients with impaired IAS functions. It has never been studied in SSc with fecal incontinence, but it does warrant consideration as it can treat Raynaud’s phenomenon as well [126]. Lastly, various questionnaires and criteria have been developed which can be helpful in diagnosing and treating GI symptoms at their earlier stages [126].

### Liver

Liver disease is rare in systemic sclerosis (SSc), affecting 1.5% of patients [147]. Of these, primary biliary cirrhosis (PBC) is the most common, accounting for up to 76.1% of these cases [148]. Thus, most of our discussion will focus on PBC in SSc. Other suggested etiologies of liver disease include nodular regenerative hyperplasia of the liver, idiopathic portal hypertension, spontaneous rupture of the liver, massive infarction of the liver, and hepatic duct obstruction related to vasculitis [149]. Rarely, liver disease may be due to autoimmune hepatitis (AIH), which, when it occurs, is almost always associated with lSSc [150,151]. Since liver disease is so rare in SSc, elevated liver enzymes should prompt consideration of concurrent hepatitis or medication toxicity [152].

SSc is the most common autoimmune disease found in patients with PBC, with a prevalence of 7–17%. SSc in its limited form accounts for 70–90% of these cases [152–155]. Antimitochondrial antibodies (AMA) found in SSc are active against the M2 autoantigen complex [156]. These antibodies were found in 85% of patients with PBC using conventional assays. Novel assays achieved sensitivities of up to 94% [157–159]. However, the role of AMA in the pathogenesis of PBC remains unclear. AMA titers in PBC-alone cases do not necessarily correlate with disease severity or rate of progression [160,161]. Anti-centromere antibody (ACA) is also found in both SSc (90% in lSSc) and PBC (30%), as well as the two in combination with a prevalence of 80% in SSc-PBC [162,163].

Overall, 1–4% of the patients with SSc are found to have co-existing PBC [153,156,164,165]. These patients tend to be older (mean age 62.7 y), have a longer SSc disease duration (mean 16.2 y), and female [153].

PBC-alone cases are usually diagnosed in patients with unexplained elevations of alkaline phosphatase (AP) of liver origin and elevated AMA (greater than titer 1:40) [166]. However, AMA positive patients with normal liver tests are now considered to also have early PBC. Liver biopsy in 29 AMA positive, non-SSc patients with normal serum bilirubin, alkaline phosphatase, and transaminases and no symptoms of liver disease showed histology that was diagnostic or compatible with PBC in 24 cases. These patients were assessed every year for 18 years; 22 patients (76%) developed symptoms of PBC and 24 (83%) had liver function tests showing cholestasis. The antinuclear antibodies, anti-gp210 and anti-sp100, are highly specific for PBC and serve as PBC markers in individuals who are AMA negative [167]. However, AMA are found in 11–13% of patients with lSSc, and up to 3% in d-SSc, and it is unclear if these patients have underlying PBC. Thus, when screening SSc patients for PBC (SSc-PBC), the more specific anti-sp100 was shown to be useful [166]. In a study of 817 patients with SSc, the combination of AMA and anti-sp100 increased the sensitivity for detection of PBC from 81.3% to 100%. Liver biopsy is only required for diagnosis when PBC-specific antibodies are absent [166].

Ursodeoxycholic acid (UDCA; 13–15 mg/kg/day) remains the treatment of choice for PBC, with liver transplantation recommended for those with late-stage disease. Other treatment modalities are of uncertain benefit and some have worsened outcomes. When added to UDCA, prednisolone and budenoside resulted in histological improvements in early stage disease. However, studies with long term follow-up are lacking. Also, sulindac and benzafibrate were shown to improve some serum liver tests in limited groups of patients with an incomplete response to UDCA [166].

Liver disease has a slower progression in patients with SSc-PBC compared with matched patients with PBC alone. Overall survival, however, is not different as there is an increase in non-liver deaths due to SSc. The rate of spontaneous bacterial peritonitis is also increased in the SSc-PBC group [155]. Nevertheless, SSc patients with early PBC (those with AMA positive test, even with normal serum bilirubin, alkaline phosphatase and transaminases) should be followed every 1–2 years for early signs and symptoms of PBC e.g. pruritus and fatigue, and for liver function test elevation [168–170]. There is currently no evidence that regular checks with liver ultrasound or transient elastography of the liver are of benefit in these patients [170].

In eleven cases of AIH, nine had lc-SSc, one had dc-SSc, and one had scleroderma-polymyositis overlap suggesting that AIH is an entitiy seen with SSc [171]. According to the European Association for the Study of the Liver (EASL), AIH should be considered in any patient with acute or chronic liver disease, particularly if hyper-gammaglobulinemia is present, and if the patient has features of other autoimmune diseases [172]. Diagnosis can be made using the simplified diagnostic criteria of the international autoimmune hepatitis group. Criteria favoring AIH include elevated gamma globulins (IgG), the presence of hepatitis on liver histology, the absence of viral hepatitis, and the presence of anti-nuclear antibodies (ANA), smooth muscle antibodies (SMA), soluble liver antigen/liver pancreas antibodies (SLA/LP), or liver/kidney microsomal antibody (LKM) [173]. Treatment of AIH should begin with prednisolone 0.5–1 mg/kg/ day. Azathioprine should be initiated at 50 mg/day whenever the serum bilirubin is below 6 mg/dL and ideally two weeks after initiation of steroid therapy. Depending on toxicity and response, azathioprine can then be increased up to a maintenance dose of 2 mg/day as prednisone is being tapered [155].

### Pancreas

Pancreatic manifestations of SSc are much less described, and often confused with the symptoms of SIBO [158]. There is no pancreatic fibrosis in SSc as originally postulated [174,175]. SSc causes exocrine pancreatic insufficiency, that is rarely clinically significant [176,177]. Pancreatic involvement maybe manifested as acute pancreatitis, painless pancreatic failure, or by a steady decrease in exocrine function punctuated by painful attacks [178]. A reduction in pancreatic secretory capacity is found in 33–61% of patients with SSc and may partially explain the diarrhea associated with SSc [179]. While tests of the stool may indicate fat malabsorption, these tests do not necessarily point to pancreatic insufficiency as the cause. Thus, antibiotics for SIBO would be tried first, and only if this fails, some suggest pancreatic enzyme replacement for the management of diarrhea [180]. If clinically indicated, the methods to assess exocrine pancreatic function include measurement of serum pancreatic iso-amylase, para-amino-benzoic acid and ultrasonography [181].

Cases of pancreatic necrosis, acute hemorrhagic pancreatitis and chronic pancreatitis have been reported in SSc due to involvement of sphincter of Odi leading to stenosis [182,183].

### Malnutrition

The decline in the nutritional status in SSc is multifactorial. Disease process, mood disturbance, and poor functional status are two factors that play an important role in the nutritional status of SSc patients [184]. A study encountered malnutrition in 55.7% of SSc patients and 20% of the reported deaths could be attributed to malnutrition [123,185].

Microstomia correlates to malnutrition risk as it was demonstrated by the Canadian Scleroderma Research Group (CSRG). CSRG utilized the “Malnutrition Universal Screening Tool” (MUST) which revealed high risk for malnutrition in 18% of the study population [186].

Due to esophageal involvement, patients experience aperistalsis and reduced lower esophageal sphincter (LES) pressure. In fact, up to 90% of patients will have some esophageal dysfunction on objective testing at some point in the disease course [187]. However, according to the CSRG study, there were no observed associations between dysphagia or reflux and malnutrition risk [186].

Gastroparesis and gastric non-compliance leads to early satiety, nausea and vomiting, bloating, and/or abdominal pain [188]. Early satiety was significantly associated with malnutrition risk in the CSRG study [186].

The small intestine is the main site of nutrient absorption along the GIT. In healthy individuals the number of bacteria in small bowel is low to prevent competition for nutrients. Natural defenses against SIBO include motility, mucosal immunity, the ileocecal valve, secretions from the intestinal walls, the pancreas and biliary tract [189].

SIBO contributes to malnutrition *via* several mechanisms including bacterial consumption of nutrients, direct damage to the intestinal mucosa, altered metabolism of carbohydrates, fats, and proteins and diarrhea. Malabsorption of micronutrients such as vitamin B12 occurs during bacterial metabolism of cobalamin that produces an inactive analog that competes with unaltered B12 for absorption. Folate and Vitamin K maintain normal or high levels due to bacterial synthesis [189].

Bacterial overgrowth may also produce symptoms that hinder food consumption. Among the most frequently reported symptoms are bloating (58.8%), abdominal pain/discomfort (54.9%), nausea (45.1%), diarrhea (27.5%), constipation (27.5%), abdominal tenderness (27.5%), vomiting (23.8%), and tenesmus (5.9%) [190].

Painful visceral and/or the cutaneous manifestations of SSc also contribute to a declining functional status. Food preparation and consumption are impaired among SSc patients due to hand involvement; the digits are affected in up to 58% of the cases during their disease [191]. Additionally, SSc also causes severe fatigue and myalgia which further contribute to a declining functional status by impairing activities of daily living such as cooking.

Due to the pervasive and progressive nature of SSc, patients suffer deterioration of their functional status which leads to mood disorders, specifically depression and poor appetite. The latter further contributes to malnutrition. As many as 56% of patients with SSc describe symptoms of major depression and 14% report dysthymia [184].

According to the North American Expert Panel, all patients with SSc should be screened for malnutrition. Their recommendations dictate that patients should be screened using a questionnaire such as the MUST questionnaire, assessment of weight loss, and body mass index (BMI). The MUST assesses malnutrition utilizing three criteria: current weight by BMI, unintentional weight loss by percentage of body mass lost, and occurrence of acute disease process that has or is expected to lead to poor nutrition for >5 days. If BMI cannot be measured, it may be estimated by measuring mid upper arm circumference (MUAC). An MUAC less than 23.5 cm estimates BMI to be less than 20 kg/m^2^ (underweight). MUAC of >32.0 cm estimates BMI to be more than 30 kg/m^2^ (obese). Weight status and unintentional weight loss are scored between 1 and 2. Acute disease causing poor nutrition receives a score of 2. Scores from each criterion are tabulated to assess malnutrition risk. A score of 0 is low risk, a score of 1 is medium and a score of >2 is high risk [192].

To evaluate the degree of gastrointestinal disease burden, the Scleroderma Gastrointestinal tract (SSc-GIT) questionnaire was developed at the University of California at Los Angeles [193]. The SSc-GIT 1.0 was first created in 2007 by identifying existing GIT questionnaires and was tested on SSc patients. The 53-item instrument was separated into 6 multi-items health-related quality of life (HRQOL) scales: “reflux/indigestion, diarrhea, constipation, pain, emotional well-being, and social functioning.” SSc-GIT 1.0 instrument was found to be reliable, and valid. In 2009, the instrument was shortened from 53 items to 34 items and named SSc-GIT 2.0; reflux/ indigestion scale was separated into reflux and bloating/distention to better identify symptoms of GERD *vs.* gastroparesis *vs.* bacterial overgrowth, and a fecal incontinence scale was added. Finally, a total GIT score was developed to identify overall burden of SSc. SSc-GIT 2.0 was found to not only reliable and valid but also be more discriminatory than self-rated individual GIT scales [194].

The use of MUST questionnaire and SSc-GIT 2.0 at regular visit intervals along with measurements of hemoglobin, vitamin A, B, folic acid and ferritin should be part of the approach to malnutrition in SSc [126]. Given the severity of symptoms and metabolic derangements because of SIBO, its management should be given priority. Fecal calprotectin has been found a useful tool in the work-up and confirmation of eradication of suspected SIBO [190]. Early referral to a gastroenterologist is crucial as these patients’ malnutrition require a multidisciplinary approach. Furthermore, appropriate imaging should be obtained if CIPO is suspected and prokinetics initiated as appropriate [126]. Given the reports of bowel perforation with octreotide, this drug should be used with caution [121,195]. If the upper GIT pathology is preventing appropriate nutrition, the next step involves the use of percutaneous tubes to allow for direct enteral delivery of nutritional formulations. Enteral tubes are preferentially placed in the jejunum after a trial of nasojejunal feeding. Even at this stage, SIBO can compromise the appropriate absorption of nutrients and parenteral nutrition might be necessary [123].

The adoption of parenteral nutrition requires a team approach that include educators, nutritionist, infection control nurse, clinical pharmacist and clinician. Monitoring include surveillance of central line associated blood stream infections, liver function test as hepatic steatosis can be seen, thrombosis and metabolic bone disease derangements [126].

There are several tools available for the assessment of sequelae encounter among SSc patients. In addition to the SSc-GIT 2.0 scoring system mentioned above, the PROMIS score and GerdQ are also available. The PROMIS score, developed by the National Institute of Health is mostly a comprehensive global patient assessment tool for assessment of disease morbidity [196]. GerdQ is used to identify the extent of the reflux symptoms that SSc patients are likely to experience at some time along their disease course [197].

## Conclusion

SSc is a rare autoimmune disorder that affects women four times more frequently than men and carries a high morbidity and mortality. It is characterized by fibrosis of multiple organs including skin, musculoskeletal system, the lungs, kidneys, heart and GI tract. Among those organs, GI tract is the most commonly affected where up to 90% of SSc patients have GI involvement. Pathogenetic mechanisms implicated in this disease include endothelial damage, vasculopathy and cytokine release triggering immune response, collagen deposition and ultimately fibrosis, the hallmark of the disease.

Antibodies against M3-R were shown to induce damage to the myenteric neurons; vascular endothelial alteration favoring fibrosis and collagen deposition have been shown to affect the neural pathways and smooth muscle activity in the GI tract resulting in the various clinical manifestations of the disease including loss of normal peristalsis, hypotonia, wall rigidity and muscular atrophy leading to paresis, dilatation, small bowel bacterial overgrowth, CIPO, diarrhea and eventually malnutrition.

The esophagus is the most commonly affected part of the GI tract, manifested by GERD, esophagitis, risk of interstitial lung disease and Barret’s esophagus, the latter necessitating close surveillance for early detection of malignant transformation.

The management options for the GI manifestations of SSc are far from ideal given the short-term efficacy and limiting side effects; these include PPI, prokinetics, antibiotics and various endoscopic interventions. Further research is needed to identify therapeutic modalities that arrest the fibrotic process early in the disease, thus preventing complications and permanent damage to vital organs. Disease modifying antirheumatic drugs have been used alone or in combination in SSc with modest benefit. Among the most promising modalities to arrest SSc tissue damage are drugs targeting TGF-β, Wnt signaling, B cell activity and hematopoietic stem cell transplantation (HSCT).
